# 2010年美国临床肿瘤学会年会热点——小细胞肺癌的研究进展

**DOI:** 10.3779/j.issn.1009-3419.2010.09.14

**Published:** 2010-09-20

**Authors:** 菊芬 蔡

**Affiliations:** 310022 杭州，浙江省肿瘤医院肿瘤内科 Department of Medical Oncology, Zhejiang Cancer Hospital, Hangzhou 310022, China

从生物学行为的角度可将肺癌分为小细胞肺癌(small cell lung cancer, SCLC)和非小细胞肺癌(non-small cell lung cancer, NSCLC)，其中SCLC约占15%左右。SCLC恶性程度高，虽然对放疗、化疗较敏感，但预后差^[1]^。本文就2010年美国临床肿瘤学会(American Society of Clinical Oncology, ASCO)年会上有关SCLC的研究进展做一综述。

## 1 SCLC的一线治疗

### 1.1 局限期SCLC的治疗

Jeong等^[2]^进行了伊力替康(irinotecan, CPT-11)联合顺铂(cisplatin, DDP)方案(IP)诱导化疗后与放疗同步治疗局限期SCLC的回顾性研究，2006年1月-2009年10月共30例患者入组，诱导化疗期间IP方案的用法为CPT-11 65 mg/m^2^和DDP 30 mg/m^2^，均为第1天和第8天使用，在放化疗同步期间IP方案的用法为CPT-11 60 mg/m^2^和DDP 30 mg/m^2^，均为第1天和第8天使用，结果显示诱导化疗及同步放化疗后的客观有效率均为100%，估计中位生存期为34.2个月，1年和2年的生存率分别为89.1%和60.9%，中位无进展生存期(progression-free survival, PFS)为11.6个月，诱导化疗期间3级和4级的中性粒细胞下降的发生率为30%，放化疗期间为15%，诱导化疗期间和同步放化疗期间3级和4级中性粒细胞减少性发热的发生率均为7%。作者认为IP方案诱导化疗后与放疗同步治疗局限期SCLC有较好的疗效和安全性，值得进一步研究。但该研究为回顾性研究，尚需前瞻性的大型Ⅲ期随机对照研究来证实，2010年版SCLC的美国国立综合癌症网络(National Comprehensive Cancer Network, NCCN)指南尚未推荐IP方案应用于局限期SCLC的治疗。

### 1.2 广泛期SCLC蒽环类药物治疗

Cornely等^[3]^进行了沙柔比星(sabarubicin)联合DDP一线治疗广泛期SCLC的研究，沙柔比星是第三代蒽环类药物，在Ⅰ期研究中，17例患者入组，3例部分缓解(partial response, PR)，当沙柔比星剂量为90 mg/m^2^及DDP 80 mg/m^2^时，5例患者中有2例出现了剂量限制性毒性(4级血小板减少1例，4级中性粒减少伴3级发热1例)，认为最大耐受剂量为沙柔比星80 mg/m^2^及DDP 80 mg/m^2^，第1天使用。在Ⅱ期研究中，沙柔比星剂量为80 mg/m^2^及DDP 80 mg/m^2^，均为第1天使用，17例男性和8例女性患者入组，其中1例患者因非治疗原因延迟治疗而被排除，1例患者疗效达完全缓解(complete response, CR)，18例患者达PR，4例患者为疾病稳定(stable disease, SD)，1例患者疾病进展(progressive disease, PD)。中位总生存期(overall survival, OS)和肿瘤进展时间(time to progression, TTP)分别为11.6个月和6.5个月，主要的不良反应发生在胃肠道(84%)和血液系统(76%)。在Ⅰ期和Ⅱ期研究中仅1例患者出现2级心力衰竭(2.4%)和1例患者出现2级心动过速(2.4%)。沙柔比星联合DDP一线治疗广泛期SCLC最大耐受剂量为沙柔比星80 mg/m^2^和DDP 80 mg/m^2^，该方案一线治疗广泛期SCLC是安全有效的。Brien等^[4]^进行了氨柔比星(amrubicin)单药或联合DDP对照足叶乙甙(VP-16)联合DDP方案(EP)一线治疗广泛期SCLC的研究。氨柔比星是人工合成的蒽环类药物和潜在的拓扑异构酶Ⅱ抑制剂，患者的入组条件为：初治、病理学证实为广泛期SCLC、WHO体力状况(performance status, PS)评分为0分-2分及根据实体瘤的疗效评价标准(Response Evaluation Criteria in Solid Tumors, RECIST)有可测量病灶。患者随机分为3组，第1组氨柔比星45 mg/m^2^第1天-第3天使用，第2组DDP 60 mg/m^2^第1天联合氨柔比星40 mg/m^2^第1天-第3天使用，第3组VP-16 100 mg/m^2^第1天-第3天联合DDP 75 mg/m^2^第1天使用，均3周为1个周期。3组患者在年龄、性别、PS等主要特征方面是平衡的，3组中位化疗周期数分别为5个周期、6个周期和6个周期。3级-4级中性粒细胞减少分别为73%、73%和69%；血小板减少分别为17%、15%和9.4%；贫血分别为10%、15%和3.1%；中性粒细胞减少性发热分别为17%、15%和14%。早期治疗相关性死亡分别为1例、3例和3例，在3组患者中均未观察到心脏毒性。在88例可评价病例中，3组的有效率分别为61%、77%和63%。氨柔比星联合DDP显示了最高的有效率。以上研究显示，沙柔比星与氨柔比星均有较好的心脏安全性和疗效，值得进一步研究。国内现正进行盐酸氨柔比星联合顺铂与依托泊苷联合顺铂化疗对照治疗广泛期SCLC的Ⅲ期临床试验(方案编号：D0750018)，我院作为该临床试验参加单位之一，现阶段试验表明在氨柔比星联合顺铂组骨髓抑制较明显，故在实际应用中需密切注意3级以上中性粒细胞减少的发生。

### 1.3 化疗联合靶向

Lubiner等^[5]^进行了CPT-11联合卡铂后舒尼替尼(sunitinib)维持治疗广泛期SCLC的Ⅱ期研究，共34例患者入组，入组条件为年龄＞18岁、初治、病理学证实为广泛期SCLC、东部肿瘤协作组(Eastern Cooperative Oncology Group, ECOG) PS评分为0分-1分、充足的器官功能和无活动性脑转移。患者接受总计最多6个周期化疗，CPT-11 60 mg/m^2^第1天、第8天和第15天，卡铂浓度-时间曲线下面积(area under the curve, AUC)按4计算，第1天使用，4周为1个周期，无疾病进展或可耐受毒性的患者接受舒尼替尼25 mg每天1次口服直到进展。化疗的中位周期数为3个周期，4例患者接受了中位4周的维持治疗，经过中位25周的随访，31例患者仍然健在，4例患者继续接受舒尼替尼维持治疗，化疗的客观有效率为47%，TTP为7.6个月。在4例接受舒尼替尼维持治疗的患者中未观察到源于舒尼替尼的3级-4级毒性。因此认为舒尼替尼作为SCLC一线双药化疗后的维持治疗具有较好的耐受性，但仍需进一步随访。由于该研究的病例数较少，需更大样本的病例来证实其安全性与有效性。Ready等^[6]^进行了舒尼替尼联合EP方案一线治疗广泛期SCLC的研究。研究因较多患者死亡及严重的骨髓抑制而中止，结果显示舒尼替尼25 mg每天口服1次，第1天-第14天使用，同时行标准EP方案化疗，将导致中性粒细胞下降时间延长和不可接受的治疗相关性死亡。化疗后使用集落刺激因子仍不能阻止严重的中性粒细胞下降和感染，因此即使在集落刺激因子支持下也不推荐使用该方案。此研究提示舒尼替尼与EP方案同时应用具有毒副作用累加效应，类似研究应慎重进行。

### 1.4 广泛期SCLC的巩固放疗

Yee等^[7]^进行了巩固放疗治疗广泛期SCLC的Ⅱ期研究，共有33例化疗后有效的病例入组，化疗后残余病灶行三维适形放疗，放疗剂量为40 Gy/15次，每天1次。结果显示28例达PR，4例达CR。最大放疗毒性为2级放射性食管炎，无治疗相关性死亡。中位TTP为8.1个月，尚未观察到中位生存期。其中11例患者有胸部复发，6例为放射野内复发，4例为系统性复发。13例为单纯远处转移，6例同时伴有远处转移和胸部复发。这一研究显示，化疗后巩固胸部放疗对化疗有效的广泛期SCLC具有较好耐受性并能降低胸部复发率。胸部放疗在局限期SCLC中有明确作用，但在广泛期SCLC中的作用有待进一步评价。

### 1.5 化疗联合生长抑素类似物

Zarogoulidis等^[8]^进行了生长抑素类似物联合化疗一线治疗SCLC的研究。共有114例(52例为局限期)初治的生长抑素受体阳性患者入组，化疗方案为紫杉醇(paclitaxel) 190 mg/m^2^联合卡铂(AUC为5.5)，均为第1天使用，最多达8个周期。化疗结束48 h后采用兰瑞肽(lanreotide)治疗，A组(30例)为兰瑞肽30 mg，B组(40例)为兰瑞肽60 mg，C组(对照组，44例)仅接受单纯化疗，A组与B组在化疗后接受兰瑞肽维持治疗，3组在局限期与广泛期SCLC的比例、年龄、PS等方面均无差异。结果显示3组患者在血液学毒性及非血液学毒性方面均无差异。A组在生存期上与B组有统计学差异，在局限期患者中A组与B组及对照组均有统计学差异。作者认为生长抑素类似物可与化疗联合治疗生长抑素受体阳性SCLC患者。

## 2 SCLC的二线治疗

### 2.1 吡铂

2010年版NCCN指南关于SCLC二线化疗的推荐为：参加临床试验、2个月-3个月内复发且PS评分为0分-2分的患者可考虑应用的药物为异环磷酰胺、紫杉醇、多西紫杉醇、吉西他滨、伊立替康、托泊替康；2个月-3个月后至6个月内复发的可考虑应用托泊替康(1级证据)、环磷酰胺联合阿霉素和长春新碱、吉西他滨、紫杉醇、多西紫杉醇、口服依托泊甙和长春瑞滨；6个月后复发的可考虑原方案。吡铂是一种为了克服铂类药物耐药而新合成的铂类化合物，Ciuleanu等^[9]^进行了吡铂(picoplatin)联合最佳支持治疗与单独支持治疗对难治性或6个月内进展的曾行一线化疗的SCLC的Ⅲ期随机研究。共有401例患者按2:1随机进入治疗组与对照组，两组中位生存期无统计学差异。在疾病进展后治疗组28%患者未接受后续化疗，对照组41%患者未接受后续化疗，在未接受该临床研究后续治疗的患者中治疗组的中位生存期为18周，对照组为14周。在PFS方面治疗组为11周，对照组为7周，两者有统计学差异。对照组的不良反应与SCLC相关，治疗组3级-4级的不良反应主要有：血小板下降为44%，贫血为29%，中性粒细胞减少为18%，无力为11%，中性粒细胞减少性发热为1%。这一研究认为吡铂可作为SCLC的二线治疗药物之一。

### 2.2 氨柔比星

Hirose等^[10]^进行了氨柔比星联合卡铂治疗难治性或复发SCLC患者的研究。患者入组条件为：PS评分为0分-2分、年龄≤75岁及充足的器官功能。氨柔比星30 mg/m^2^第1天、第2天和第3天，卡铂按AUC=4计算，第1天使用，3周为1个周期。2005年6月-2009年4月，共有28例患者入组，局限期3例，广泛期25例。结果显示有效率为35.7%，敏感复发的患者有效率为63.6%，中位生存期为184天。3级-4级中性粒细胞下降为86%，血小板下降为46%，贫血为61%，3级感染为7%，无治疗相关性死亡。该方案被认为对难治性或复发的SCLC患者是安全有效的。笔者认为，氨柔比星与卡铂均有明显骨髓抑制作用，实际应用中需高度重视可能出现的骨髓抑制。

### 2.3 ABT-263

ABT-263是Bcl-2蛋白抑制剂，Rudin等^[11]^进行了ABT-263治疗复发SCLC的临床研究，2009年6月-2009年12月，共有39例患者入组，ABT-263每天口服325 mg(前7天导入期剂量为150 mg)，3周为1个周期。21例患者因疾病进展而停止，4例退出研究，6例患者因不良反应减量，11例患者出现严重不良反应，主要的不良反应为腹泻(43%)、背痛(43%)和血小板减少(29%)。4例患者因不良反应而中断研究，其有效性的评价正在进行中。

### 2.4 拓扑替康联合贝伐单抗

Waterhouse等^[12]^进行了拓扑替康联合贝伐单抗二线治疗SCLC的Ⅱ期临床研究。拓扑替康2.3 mg/m^2^第1、2、3、4和5天使用，贝伐单抗15 mg/kg第1天使用，21天为1个周期。所有患者均需有充足的器官功能，ECOG PS评分为0分-2分，计划治疗8个周期或出现疾病进展或不能耐受的毒性。50例患者入组，6例患者仍在治疗中，44例患者已中止，其中24例由于疾病进展，10例由于不良反应。中位PFS及OS分别为17.4周和31.6周，有效率为10%。3级和4级中性粒细胞下降和血小板减少分别为44%和50%，未发生3级和4级的蛋白尿和高血压，非血液学毒性主要为原发性恶心、腹泻、呕吐、虚弱和疲劳。有5例非疾病相关死亡：血小板减少、上消化道出血、呼吸衰竭、肺炎和败血症。提高3个月PFS的目标未达到，这种联合方案的边缘获益不能完全排除。贝伐单抗在SCLC中的应用有待进一步评价。

## 3 其它

Foster等^[13]^进行了肿瘤有效率和PFS作为广泛期SCLC OS潜在替代终点的研究。共有870例初治患者入组，分别来自6个单中心的临床试验(274例)和3个多中心的临床试验(596例)。结果显示，PFS与OS有很强的相关性，PFS非常有希望作为OS的替代，但仍需更多Ⅲ期随机临床试验来证实。PFS作为OS的替代终点有利于快速而有效地评价治疗广泛期SCLC的药物。Gadgeel等^[14]^进行了美国底特律肺癌脑转移患者流行病学研究，共有75 564例肺癌患者。结果显示，SCLC发生脑转移的比例为25.1%，SCLC中有脑转移的患者死亡风险并未明显高于无脑转移患者。

Hltermann等^[15]^进行了循环肿瘤细胞作为SCLC预后因子的研究。所有SCLC患者均经病理证实，且进行最多4个周期的EP方案化疗。化疗前，第2周期化疗前，化疗结束后分别抽取20 mL血液，72 h内在同一中心实验室分析循环肿瘤细胞，根据患者循环肿瘤细胞水平分为预后良好及不良组，共有41例患者入组，根据年龄、性别、PS评分及分期进行校正。结果显示，基础的循环肿瘤细胞水平和一线化疗后早期下降是其独立的预后因子。

## 4 小结

IP方案诱导化疗后与放疗同步治疗局限期SCLC具有较好的疗效和安全性，值得进一步评价。新一代蒽环类药物包括沙柔比星与氨柔比星在广泛期SCLC的一线治疗中有较好疗效与心脏安全性，值得进一步开展临床研究。舒尼替尼与EP方案同时应用具有较大毒性，而舒尼替尼是否可用于维持治疗有待进一步评价。广泛期SCLC的巩固放疗有降低胸部复发率的可能，生长抑素类似物与化疗联合对于生长抑素受体阳性的SCLC患者具有较好疗效。新合成的铂类化合物吡铂及新合成的蒽环类药物氨柔比星用于SCLC二线治疗具有较好的疗效和安全性。ABT-263和贝伐单抗在SCLC二线治疗中的作用尚不够明确。PFS有望作为广泛期SCLC OS的潜在替代终点，基础循环肿瘤细胞水平和一线化疗后早期下降是SCLC的预后因子。
